# A Common Cortical Circuit Mechanism for Perceptual Categorical Discrimination and Veridical Judgment

**DOI:** 10.1371/journal.pcbi.1000253

**Published:** 2008-12-26

**Authors:** Feng Liu, Xiao-Jing Wang

**Affiliations:** 1 Department of Physics, Nanjing University, Nanjing, People's Republic of China; 2 Department of Neurobiology and Kavli Institute for Neuroscience, Yale University School of Medicine, New Haven, Connecticut, United States of America; University College London, United Kingdom

## Abstract

Perception involves two types of decisions about the sensory world: identification of stimulus features as analog quantities, or discrimination of the same stimulus features among a set of discrete alternatives. Veridical judgment and categorical discrimination have traditionally been conceptualized as two distinct computational problems. Here, we found that these two types of decision making can be subserved by a shared cortical circuit mechanism. We used a continuous recurrent network model to simulate two monkey experiments in which subjects were required to make either a two-alternative forced choice or a veridical judgment about the direction of random-dot motion. The model network is endowed with a continuum of bell-shaped population activity patterns, each representing a possible motion direction. Slow recurrent excitation underlies accumulation of sensory evidence, and its interplay with strong recurrent inhibition leads to decision behaviors. The model reproduced the monkey's performance as well as single-neuron activity in the categorical discrimination task. Furthermore, we examined how direction identification is determined by a combination of sensory stimulation and microstimulation. Using a population-vector measure, we found that direction judgments instantiate winner-take-all (with the population vector coinciding with either the coherent motion direction or the electrically elicited motion direction) when two stimuli are far apart, or vector averaging (with the population vector falling between the two directions) when two stimuli are close to each other. Interestingly, for a broad range of intermediate angular distances between the two stimuli, the network displays a mixed strategy in the sense that direction estimates are stochastically produced by winner-take-all on some trials and by vector averaging on the other trials, a model prediction that is experimentally testable. This work thus lends support to a common neurodynamic framework for both veridical judgment and categorical discrimination in perceptual decision making.

## Author Summary

In daily life, we constantly face two types of perceptual decisions: to identify an object feature (what is the speed of that car?) or to discriminate the same feature among two or more possible categories (is that car going faster than the speed limit?). These decision processes appear to involve very different computations: while identification relies on an analog judgment, categorical discrimination is based on a comparison of the object feature with discrete options. Do they engage entirely separate brain mechanisms? In this work, we showed that these two types of decision making can be instantiated by a single cortical circuit. We used a continuous recurrent network model to simulate two monkey experiments in which subjects were required to make either a two-alternative choice or a veridical judgment about the direction of random-dot motion. The model reproduced salient experimental observations and makes testable predictions. The results demonstrate that a common cortical circuit can perform both categorical discrimination and veridical judgment. Conceptually, this work supports the notion that a cortical circuit endowed with reverberatory dynamics can fulfill multiple cognitive functions such as working memory and decision making.

## Introduction

Perceptual judgments involve detection, identification and discrimination of objects in a sensory scene [1],[2]. Given an ambiguous visual motion pattern, for instance, a subject may be asked to detect whether a net motion direction is present or absent [3], to identify the motion direction as an analog quantity [4], or to discriminate the motion direction between two options (e.g., left or right) [5]. Using the strategy of single-unit recording from behaving monkeys, neurophysiologists have begun to uncover neuronal activity that is linked to such perceptual judgments (for reviews, see [6]–[11]). In monkey experiments using perceptual discrimination tasks, neural correlates of decision making have been observed in the parietal [12],[13], premotor [14]–[16] and prefrontal [17],[18] cortical areas. Experimental observations have inspired the advance of neural circuit models which suggest that recurrent (attractor) network dynamics can underlie temporal integration of sensory information (accumulation of evidence) and decision formation [18]–[25].

Focusing on categorical discrimination, those neural circuit models as well as abstract ramp-to-threshold models [26]–[30] are typically endowed with a simple architecture consisting of discrete neural pools, selective for categorical alternatives. Therefore, they are inadequate for exploring perceptual identification that requires neural representation of analog quantities, such as motion direction that can be an arbitrary angle between 0° and 360°. On the other hand, probabilistic estimation of an analog stimulus feature has been studied from the perspective of optimal population coding [2],[31],[32]. These studies centered on optimal algorithms for reading out a stimulus feature from sensory neural populations, such as inferring the orientation of a visual stimulus from neural activity in the primary visual cortex [33] and the direction of a motion stimulus from activity profiles across the middle temporal visual area (MT) [2]. However, such probabilistic inference is believed to occur in higher-order cortical areas downstream from primary sensory areas, and the underlying circuit mechanism remains unclear. In particular, it is unknown whether probabilistic estimation and categorical discrimination engage distinct decision processes or can be realized by a shared neural circuit mechanism.

**Figure 1:**
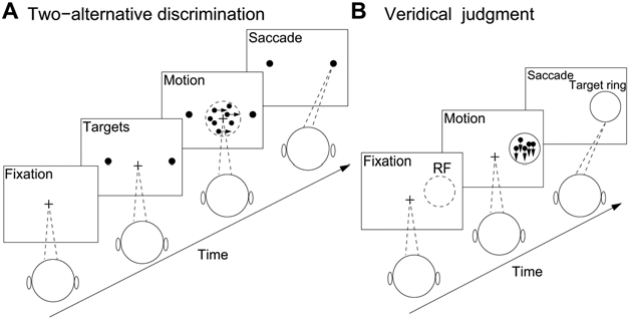
Schematic depiction of two monkey experiments that were simulated by the continuous recurrent network model. (A) Reaction-time version of a two-alternative forced-choice direction discrimination task. A trial began when the monkey fixated a point on the display monitor. Two choice targets then appeared in the periphery. One was within the response field (RF) of the recorded neuron, and the other was in the opposite hemisphere. After a delay, a dynamic random-dot display appeared, where a fraction of dots moved coherently toward one of the two targets while the others moved randomly in all other directions. The monkey was allowed to make a saccadic eye movement toward a target at any time when it was ready. (B) Direction identification task. After fixation, a random-dot motion stimulus appeared inside a target ring and lasted 1 s. When the fixation point was extinguished, the monkey made a saccadic eye movement to the location on the target ring toward which the dots had moved. On some trials, electrical stimulation was simultaneously applied to MT neurons.

In the present work, we investigated this outstanding question using a continuous recurrent network model of spiking neurons, which was initially proposed for spatial working memory [34]. We applied this model to the simulation of two monkey experiments using random-dot visual motion stimuli. In a two-alternative forced-choice direction discrimination task (Figure 1A), the monkey was trained to discriminate the motion direction by making a saccadic eye movement to one of two peripheral choice targets [12],[13],[35]. It was found that ramp-like spiking activity of neurons in the lateral intraparietal cortex (LIP) is correlated with the monkey's choice. By contrast, in a direction identification task (Figure 1B), the monkey was required to report veridically its perceived direction of motion in the visual stimulus [4]. On some trials, electrical stimulation was applied simultaneously to MT neurons when the monkey viewed the random-dot display. Microstimulation could bias the monkey's judgments toward the preferred direction of MT neurons at the microstimulation site [4],[36]. It was argued that both vector-averaging and winner-take-all algorithms might contribute to the interpretation of activity profiles of MT neurons. But [4] collected only behavioral data and did not record neural activity in MT or downstream cortical areas. Thus, the neural mechanism for veridical judgments about the motion direction remains unknown.

Here we show that the continuous recurrent network model is capable of reproducing salient observations from both experiments. Our results suggest that both categorical discrimination and veridical judgment can be subserved by a common cortical circuit endowed with reverberatory dynamics.

## Materials and Methods

### Network Architecture

Our model is designed to simulate two perceptual decision tasks in which the decision is about the net direction of a random-dot motion stimulus. Since the directional angle is a one-dimensional quantity, we used a continuous network model in which each neuron is selective for a motion direction, from 0° to 360°. Our model network does not directly map onto LIP, in which neurons have response fields in a two-dimensional visual space. However, our model is adequate for simulating the two tasks, and we do not anticipate that a two-dimensional version of our model would behave in qualitatively different ways.

**Figure 2:**
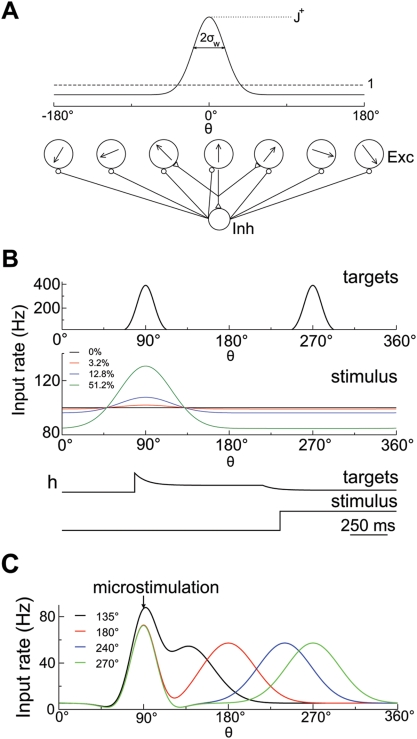
Network architecture and input signals. (A) Schematic illustration of network structure. The network is composed of 2,048 pyramidal cells and 512 interneurons. Excitatory cells are labeled and arranged by their preferred motion directions (from 0° to 360°). The connectivity between pyramidal cells is structured, and the synaptic strength is a Gaussian function of the difference between their preferred directions (solid curve). Connections to or from inhibitory interneurons are broad. (B) Spatial profile and time course of input rates in the direction discrimination task. External inputs to the network from two targets and the motion stimulus are separately modeled as excitatory synaptic currents mediated by AMPA receptors, with presynaptic spikes emitted based on Poisson processes. Poisson rates are depicted in the figure as a function of preferred directions of neurons and time: the maximum input rate from two targets, the input rate from the motion stimulus for four different motion strength, and their corresponding time courses, respectively (from top to bottom). For the target input, the effects of spike-rate adaptation and divided attention upon stimulus onset are included. (C) Spatial profile of input rate in the direction identification task. The inputs from both the motion stimulus and microstimulation are modeled as excitatory synaptic currents. The profiles of Poisson rate are shown for four different stimulus directions with the microstimulated direction fixed at 90°.

The model network is composed of *N*_E_ pyramidal cells and *N*_I_ interneurons. The network architecture is consistent with a columnar organization [34],[37]. Cells are spatially distributed on a ring according to the motion direction to which they are most sensitive (Figure 2A). Each neuron is labeled by its preferred direction *θ_i_*, which is uniformly distributed between 0° and 360°. Simulations were done with *N*_E_ = 2048 and *N*_I_ = 512.

### Neurons and Synapses

Both pyramidal cells and interneurons are described by leaky integrate-and-fire neurons and are characterized by six parameters [34]: the membrane capacitance *C*_m_, the leak conductance g_L_, the resting potential *E*_L_, the threshold potential *V*_th_, the reset potential *V*_reset_, and the refractory time *τ*_ref_. The values used were: *C*_m_ = 0.5 nF, *g*_L_ = 25 nS, *E*_L_ = −70 mV, *V*_th_ = −50 mV, *V*_reset_ = −59 mV, and *τ*_ref_ = 2 ms for pyramidal cells; *C*_m_ = 0.2 nF, *g*_L_ = 20 nS, *E*_L_ = −70 mV, *V*_th_ = −50 mV, *V*_reset_ = −59 mV, and *τ*_ref_ = 1 ms for interneurons. Below *V*_th_, the membrane potential *V_i_*(*t*) of cell *i* obeys the following equation:
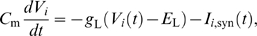
where *I_i_*_,syn_ represents the total synaptic current flowing into the cell.

The network is endowed with pyramidal-to-pyramidal, pyramidal-to-interneuron, interneuron-to-pyramidal, and interneuron-to-interneuron connections (Figure 2A). For the sake of simplicity, only the connectivity between pyramidal cells is structured. Recurrent excitatory currents are mediated by AMPA receptors (AMPARs) and NMDA receptors (NMDARs), while inhibitory currents are mediated by GABA_A_ receptors (GABA_A_ Rs). External excitatory inputs include those from MT neurons, which represent visual motion stimuli and electrically elicited directional signals. When simulating the categorical discrimination task, additional inputs represent the presentation of choice targets. All neurons also receive background synaptic input mimicking spontaneous activity outside the local network. In simulations, all these external currents are mediated exclusively by AMPARs.

The total synaptic current in pyramidal cell *i* is given by
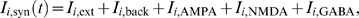
where
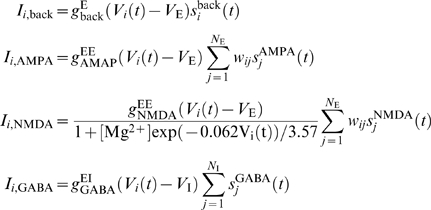
with *V*_E_ = 0 mV and *V*_I_ = −70 mV. *I_i_*_,back_ represents background synaptic input. *I_i_*_,AMPA_ and *I_i_*_,NMDA_ denote recurrent excitatory inputs, while *I_i_*_,GABA_ represents recurrent inhibitory input. The maximum synaptic conductances are denoted by 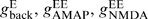 (pyramidal-to-pyramidal), and 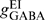 (interneuron-to-pyramidal), respectively. We shall describe *I_i_*_,ext_ in the following sections.

For interneuron *i*, the total synaptic current is described similarly except for *I_i_*_,ext_ = 0 as well as different synaptic conductances 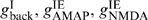 (pyramidal-to-interneuron), and 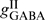 (interneuron-to-interneuron).

The synaptic strength between two pyramidal cells *i* and *j* depends on the difference between their preferred directions and is described as 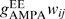 or 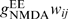 with 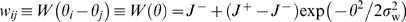. If *θ*>180°, it is set to *θ*−360, and if *θ*<−180°, it is set to *θ*+360. This is done to satisfy the periodic boundary condition, which is also imposed on the following Equations 2–5. Note that *W*(*θ*) is normalized as
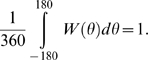

*W*(*θ*) with 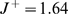 and *σ_w_* = 18° is shown in Figure 2A (solid curve).

The gating variables, i.e., the fractions of open channels, are described as follows. The AMPA (external and recurrent) synaptic variable obeys the following equation:
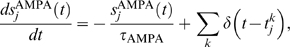
(1)where the decay time constant was set to *τ*_AMPA_ = 2 ms, and the sum over *k* represents a sum over spikes emitted by presynaptic neuron *j* [19]. In the case of background noise, 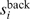 also obeys Equation 1, where spikes are emitted based on a Poisson process with a rate of 1.5 KHz independently from cell to cell. The maximum conductances were set to 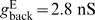 and 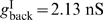. NMDA currents have a voltage dependence that is controlled by the extracellular magnesium concentration, [Mg^2+^] = 1 mM. Thus, the NMDA channel kinetics are modeled as
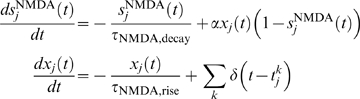
with *τ*_NMDA,decay_ = 100 ms, *α* = 0.5 ms^−1^, and *τ*_NMDA,rise_ = 2 ms [19]. The GABA synaptic variable obeys the following equation:
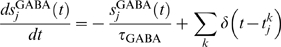
with *τ*_GABA_ = 10 ms. All synapses have a latency of 0.6 ms.

In simulations of the discrimination task, the maximum recurrent synaptic conductances (in *µ*S) were taken as 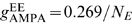, 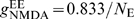, 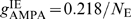, 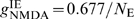, 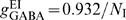, and 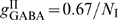. These conductances are scaled inversely proportionally to the number of pyramidal cells and of interneurons, respectively. This is to keep the total synaptic conductances unchanged when network size is varied. With these parameter values, NMDAR channels contribute 85% to recurrent excitatory charge entry at a holding potential of −65 mV. To simulate the identification task, we decreased the conductance values except 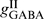. Meanwhile, we increased the ratio of 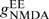 to 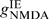 and of 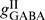 to 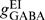 so that the overall recurrent inhibition is decreased. The following values were used: 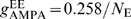, 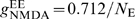, 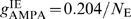, 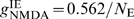, 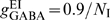, 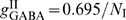 as well as 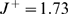 and *σ_w_* = 14°. In this case, NMDAR channels contribute 83.5% to recurrent excitatory charge entry at a holding potential of −65 mV. Three features are worth noting. First, recurrent excitation is taken to be primarily mediated by NMDARs [38]. Second, the network is dominated by recurrent inhibition [34]. Third, neurons receive a large amount of background noise.

### Two-Alternative Direction Discrimination Task

To simulate a two-alternative direction discrimination task [13],[35], the presentation of two choice targets at *θ*_1_ and *θ*_2_ is modeled through selective synaptic input to the pyramidal cells whose preferred directions are close to either *θ*_1_ or *θ*_2_. The random-dot motion stimulus is represented by MT neurons, which project to LIP. Therefore, the external input to pyramidal cell *i* is assumed to be *I_i_*_,ext_(*t*) = *I_i_*_,tar_(*t*)+*I_i_*_,stim_(*t*) with
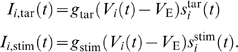


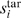
 and 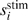 obey Equation 1, with spikes discharged according to Poisson processes with rates 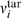 and 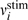, respectively.


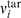
 depends on the preferred direction *θ_i_* of each cell and varies with time; it is described as
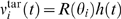
with
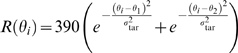
(2)

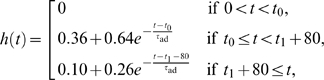
where *t*_0_ and *t*_1_ represent the onset times for the targets and the stimulus, respectively. The function *h*(*t*) models the spike-rate adaptation of upstream neurons encoding the targets and the presumed divided attention upon stimulus onset. The adaptation time constant *τ*_ad_ was set to 80 ms. Upon the stimulus onset, the strength of target input is assumed to be reduced, presumably resulting from a cross inhibition between upstream neurons separately signaling the motion stimulus and the targets, or because the subject's covert attention is shifted from the targets to the stimulus. Consequently, the neural activity decreases momentarily, resembling a brief ‘dip-and-rise’ in firing rate of LIP neurons. We used the following values: *θ*_1_ = 90°, *θ*_2_ = 270°, *σ*_tar_ = 13°, *t*_0_ = 500 ms, *t*_1_ = 1300 ms, and *g*_tar_ = 12 nS (Figure 2B). The specific parameter values in *R*(*θ_i_*) and *h*(*t*) are not so important, provided that the input from the targets is sufficiently strong to trigger high neural activity before stimulus presentation.

Based on the tuning curves of MT neurons during the presentation of a random-dot display [39], 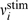 is modeled as
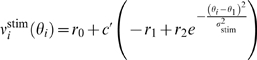
(3)with *c*′ (0≤*c*′≤1) denoting motion strength and *θ*_1_ the direction of coherent motion. We used the following values: *r*_0_ = 100 Hz, *r*_1_ = 30 Hz, *r*_2_ = 90 Hz, *σ*_stim_ = 40°, and *g*_stim_ = 5.9 nS (Figure 2B). Note that there is a latency for visual signals to arrive in LIP, which was assumed to be 200 ms [29],[35].

### Direction Identification Task

The simulations used the same protocol as in [4]. Pyramidal cells in the model circuit receive excitatory synaptic input from MT neurons representing both the motion stimulus and the electrically evoked directional signal. MT activity is broadly tuned to visual motion stimuli, characterized by tuning curves with a typical width at half-height of ∼90° [39]–[42]. On the other hand, we assume that microstimulation activates a much narrower range of MT neurons and also evokes lateral inhibition from interneurons. As a result, the external input is described as
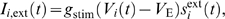
where 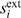 obeys Equation 1, with spikes emitted based on a Poisson process with a rate *μ_i_*. In the presence of only the visual stimulus,
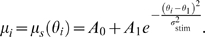
(4)


In the presence of microstimulation alone,
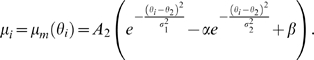
(5)


As a first-order approximation, *μ_i_* = *μ_s_*(*θ_i_*)+*μ_m_*(*θ_i_*) in the presence of both the visual stimulus and microstimulation, which are delivered simultaneously and last a fixed duration of 1 s. Equation 4 is similar to Equation 3. The second term on the right-hand side of Equation 5 is to mimic lateral inhibition from interneurons; the third term is to ensure *μ_m_* positive.

The directional angles *θ*_1_ and *θ*_2_ denote the coherent motion direction in the random-dot display and the preferred direction of MT neurons at the microstimulation site, respectively. We assume *A*_0_ = 7−3.5*c*′ and *A*_1_ = 49*c*′ (in units of Hz) with *c*′ being the stimulus coherence level. As in the experiment, *c*′ was always set to 80% representing a vivid suprathreshold stimulus unless specified otherwise. This is so because the experimental study aimed to investigate the interaction between this suprathreshold motion stimulus and microstimulation at varying angular distances. Other parameter values were chosen so that the maximum firing rate of cells at stimulus offset is comparable when microstimulation or the visual stimulus is presented alone. The values used were: *A*_2_ = 86.8 Hz, *α* = 0.25, *β* = 0.05, *σ*_1_ = 21°, *σ*_2_ = 33°, *σ*_stim_ = 40°, *θ*_2_ = 90°, and *g*_stim_ = 6.1 nS. *θ*_1_ varied with trials.

The angular difference Δ*θ* = |*θ*_2_−*θ*_1_| can be used to classify neural activity. For a small Δ*θ*, there is a significant overlap between the two inputs, *μ_s_* and *μ_m_*, and there is a relatively large value in between two peaks (Figure 2C). For a large Δ*θ*, the two inputs are nearly independent of each other.

### Readout of the Direction Judgment

For both the direction discrimination and identification tasks, we used the same measure to read out direction judgment. It is determined by a population vector scheme as follows [43]:
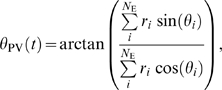
where *r_i_* is the instantaneous firing rate of cell *i*, of which the preferred direction is *θ_i_*. Especially, the value of *θ*_PV_ at stimulus offset is denoted by *θ*_E_, which represents a direction estimate on individual trials. *r_i_* is calculated as follows. For each time window of 40 ms (with a sliding window being 5 ms), the total spike number is counted and divided by the time window.

For the reaction-time version of the discrimination task, we also read out decision time based on threshold crossing of neural population firing rates. Specifically, we calculated the instantaneous population firing rates, *r*_1_ and *r*_2_, of two neural pools separately centered at *θ*_1_ and *θ*_2_, each consisting of 140 cells and spanning 360°×(140/2048)∼24°. That is, each pool consists of cells with their preferred directions within ∼±12° around *θ*_1_ or *θ*_2_. The time bin was 40 ms, and a sliding window of 5 ms was used to smooth data. Decision time is calculated by assuming that a decision is made whenever *r*_1_ or *r*_2_ first reaches a prescribed threshold, which was set to 57 Hz to fit behavioral data. Decision times can be compared with experimentally recorded reaction times by adding a non-decision response time ∼70 ms (i.e., the additional time it takes for a monkey to generate a saccadic eye movement after a choice is made).

### Numerical Method

**Figure 3:**
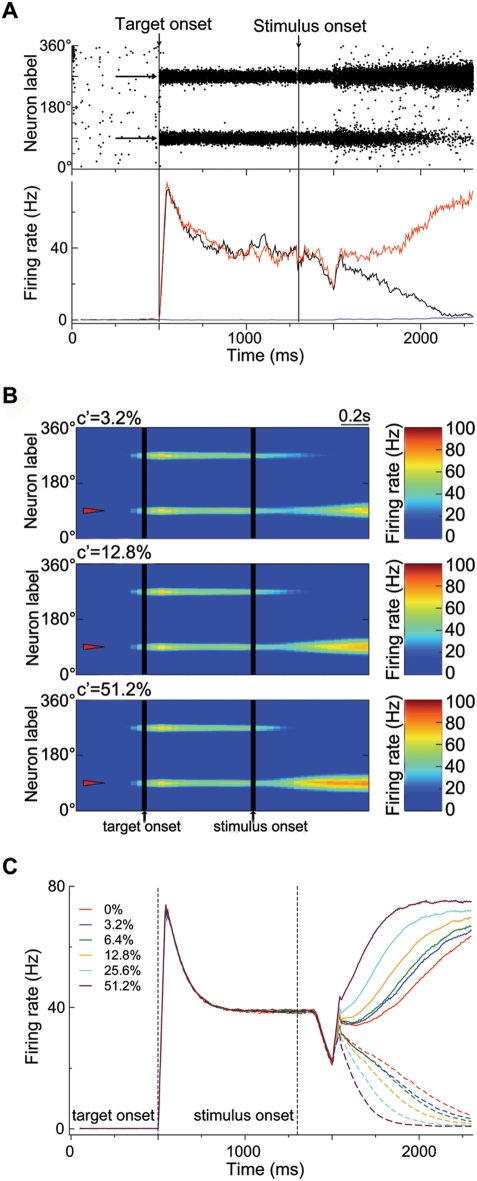
Network activity during the direction discrimination task. (A) (Top) Spatiotemporal firing pattern of pyramidal cells with the stimulus at zero coherence. *x*-Axis, time; *y*-axis, cells labeled by their preferred directions. Two targets are separately presented at 90° and 270° (indicated by arrows). The targets and the motion stimulus are presented at 500 ms and 1,300 ms, respectively. But there is a latency (about 200 ms) for the visual signal to reach LIP. (Bottom) Time course of the population firing rates for the two neural pools, each consisting of 140 neurons and separately centered at 90° (*r*_1_, black) and 270° (*r*_2_, red), and for the neurons whose preferred directions are at least 26° away from 90° and 270° (blue), respectively. (B) Network activity patterns shown with a color-coded firing rate map for three coherence levels. The coherent motion direction is 90° (indicated by triangles). (C) Time course of population firing rates *r*_1_ (solid curves) and *r*_2_ (dashed curves), averaged over 1,000 correct trials, for various coherence levels. See Results for detailed description.

The trial-averaged population firing rates were obtained by averaging over 1000 correct trials (Figure 3C). Moreover, to visualize network activity, spatiotemporal maps of firing rate are shown in Figure 3B. A spike time rastergram for all pyramidal cells was smoothed with a sliding window both in time (50 ms) and along the neural population (10 neurons). The resulting firing rate was color coded. The integration method used is a modified second-order Runge-Kutta algorithm [44], with a time step of 0.02 ms.

## Results

We will first report model simulations of the categorical discrimination task [13] and assess how well the model reproduces the monkey's performance as well as LIP activity that appears to reflect the decision computation. We will then use the same model to simulate the direction identification task involving the microstimulation of MT [4]. We will examine how a continuous recurrent circuit, endowed with strong reverberatory dynamics, can integrate sensory information and make categorical choices in the discrimination task or instantiate both the winner-take-all and vector-averaging mechanisms for direction judgments in the identification task.

### Two-Alternative Forced-Choice Direction Discrimination Task 

Graded Ramping Neural Activity and Categorical Competition

Model simulations used the same protocol as in the reaction-time version of a two-alternative direction discrimination task [13]. Figure 3A displays typical network activity in response to both two targets and a random-dot motion stimulus at zero coherence. The network activity is monitored by plotting its spatiotemporal firing pattern (*upper panel*). A trial begins with the network in a resting state in which cells exhibit low spontaneous firing. Two targets are then separately presented at *θ*_1_ (90°) and *θ*_2_ (270°), instructing the network two choice options. In response, two neural pools separately centered around *θ*_1_ and *θ*_2_ show persistent elevated activity, with neural discharges quite asynchronous. Thus, the profile of network activity exhibits two symmetric ‘bumps’ separately centered at *θ*_1_ and *θ*_2_. That is, there is no winner-take-all competition in the symmetric state. This has also been observed in [23] and can be understood as follows. In our model, recurrent excitation is dominated by the NMDARs-mediated current, which saturates at high firing rates [38]. The winner-take-all mechanism requires not only global inhibition but also recruitment of synaptic excitation. This recurrent excitation saturates at (symmetric) high firing rates, and thus no winner-take-all occurs.

Upon the onset of motion stimulus, neural activity decreases transiently owing to a reduced efficacy of target input (see Methods). The biological origin of this reduction is currently unknown; possible scenarios include a cross inhibition between upstream neurons separately signaling the targets and the motion stimulus and that the subject's covert attention may be shifted from the targets to the stimulus. After the visual signal reaches the decision circuit (with a latency of 200 ms), the two neural pools integrate the signal and compete against each other through shared inhibitory feedback from interneurons. Eventually, one neural pool wins the competition and increases its activity, while the other's activity is greatly suppressed, leading to a categorical choice. Note that winner-take-all competition occurs even when the stimulus input is uniform across the network. This is interpreted as follows. The symmetric state with high firing rates is stable only for sufficiently strong inputs. It disappears and is replaced by asymmetric states (with one of the two bumps growing while the other shrinking) when the target input is reduced to lower levels after stimulus onset, similar to the behavior of a model network composed of discrete neural pools [23].

The decision process can be revealed by showing the time course of population firing rates, *r*_1_ and *r*_2_, of the two neural pools separately centered around *θ*_1_ and *θ*_2_ (see Methods). In response to target presentation, *r*_1_ and *r*_2_ initially display a drastic increase followed by an adaptation to ∼40 Hz (Figure 3A, lower panel), resembling the LIP response to target presentation [12],[13],[35]. After the motion stimulus is delivered, both *r*_1_ and *r*_2_ first decrease and then rise together to nearly the same level as before stimulus onset. Such a dip-and-rise has been widely observed in experiments [13],[35],[45],[46]. Afterwards, *r*_1_ and *r*_2_ begin to diverge over time, with *r*_2_ climbing up while *r*_1_ decaying down in this example. This subserves the formation of a binary decision. A choice is made when *r*_2_ reaches a prescribed threshold. Throughout the decision process, there is a dynamic balance between recurrent excitation and inhibition, as the activity of interneurons builds up in parallel with that of winning pyramidal cells (data not shown). This excitation-inhibition balance is important for ensuring network stability and, together with background synaptic noise, contributes to stochastic network dynamics. Given the stimulus at zero coherence, this stochasticity determines the choice outcome on any given trial, and thus the decision is at chance level across trials.

Figure 3A also displays the time course of the mean firing rate of the pyramidal cells which are not activated directly by the two target inputs (blue curve). After the presentation of two targets, since the two activated neural pools (in the “bumps”) excite interneurons, which in turn send feedback inhibition globally to the entire network, those pyramidal cells show a suppressed activity compared to the spontaneous state. After the visual stimulus reaches the decision network, those cells also receive an extra external activation (e.g., the motion stimulus is uniform at zero coherence). Meanwhile, the feedback inhibition decreases because of the drop of neural activity in one of the two bumps. These two factors combined lead to the increase of firing activity of those cells.

In the monkey experiment, coherence level or motion strength *c*′ refers to the fraction of dots that move coherently in one particular direction (e.g., 90°) while the others move randomly in all other directions with a uniform distribution in the random-dot display. This is implemented in the model as bell-shaped input profiles (see Figure 2B), which mimic the activity profiles of MT neurons at different coherence levels [39]. In Figure 3B is shown the network activity on single trials with stimuli at nonzero coherence levels. After two targets are presented, two bumps separately develop around *θ*_1_ and *θ*_2_. Since the targets exist throughout the trial, they ‘instruct’ the network two choice options and always exert an influence on the decision process. After stimulus onset, neural activity first decreases briefly and then rises. Furthermore, there is a transition from the symmetric state to the asymmetric state, where one bump eventually becomes predominant over the other. This transition occurs faster with increasing coherence level.

Figure 3C displays the time course of population firing rates *r*_1_ and *r*_2_, averaged over correct trials, for different *c*′ values. Immediately after stimulus onset, there is a dip-and-rise in population activity, which is independent of motion strength, similar to the observation from LIP neurons [13],[35]. About 200 ms after stimulus onset, *r*_1_ and *r*_2_ begin to diverge and vary in a ramp-like pattern, which underlies the network's temporal integration of sensory inputs. The ramping activity is faster with a larger slope at higher *c*′. Moreover, at lower *c*′, immediately after the dip-and-rise, the firing rate of the winning pool shows a momentary plateau for ∼100 ms before it ramps up (see *red*, *green and blue solid curves*). This biphasic behavior (i.e., plateau-and-ramp) has been observed in LIP activity [30],[35] and in our previous model [21]. Therefore, the graded ramping activity reflects the quality of sensory evidence, and the ultimate divergence in spiking rate of competing neural pools gives rise to a choice. Figure 3C is remarkably similar to the LIP activity observed experimentally (see Figure 7A in [13] and Figure 5A in [35]). Note that only one neuron was recorded at a time in the experiment. Nevertheless, the simulation results can be compared with the physiological data, if the activity of the winning (respectively losing) pool is mapped onto that of an LIP neuron on trials when the monkey's choice is toward (respectively away from) its preferred direction. Therefore, the model reproduces the salient characteristics of LIP activity correlated with perceptual decision making.

### Psychometric Function and Decision Time

**Figure 4:**
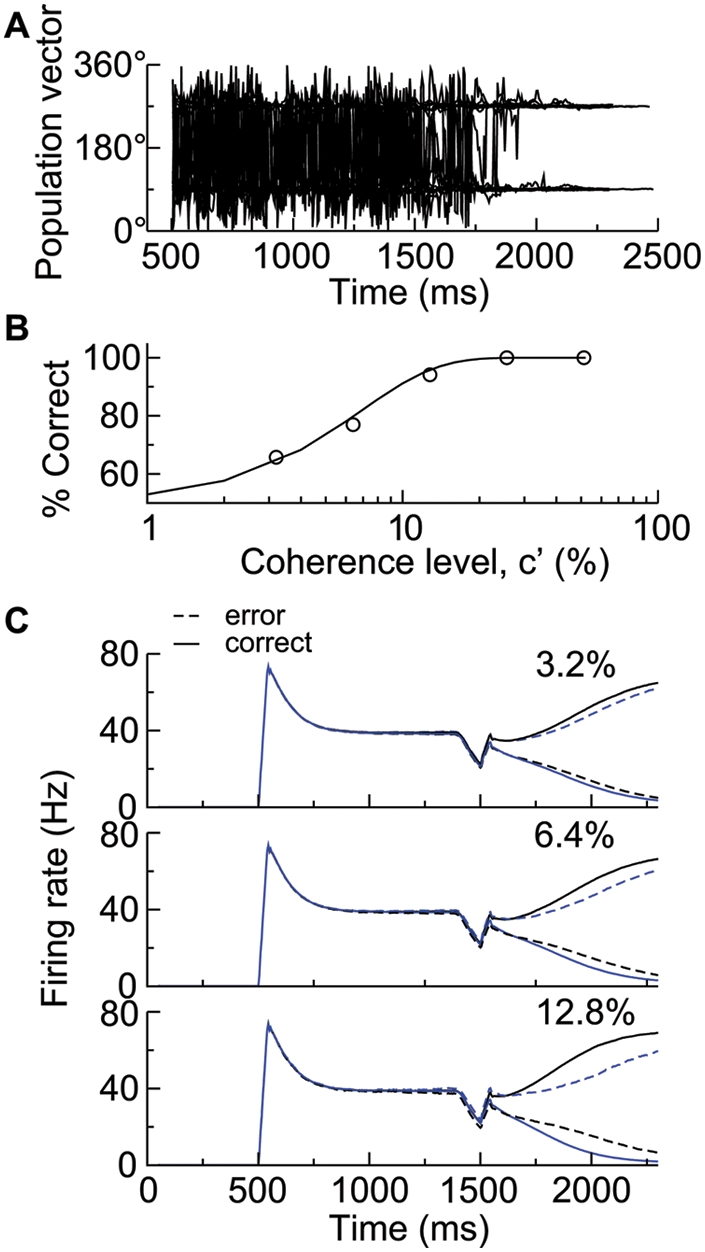
The network's performance and population activity during the direction discrimination task. (A) Time course of population vector. Twenty traces are shown with the stimulus at zero coherence. (B) The probability of correct choices versus motion strength. Data (circle) are fitted by a Weibull function with *α* = 6.85% and *β* = 1.45 (solid curve). (C) Time course of population firing rates *r*_1_ (black) and *r*_2_ (blue), averaged over correct (solid curves) and error (dashed curves) trials, respectively, for three coherence levels.

The model network's performance is measured as follows. For each *c*′ value, simulations are run thousands of times, and the choice on each trial is read out according to which of the two neural pools wins the competition or based on the population vector *θ*_PV_. Figure 4A shows 20 traces of *θ*_PV_ with the stimulus at zero coherence. Clearly, when either population firing rate first reaches a threshold (57 Hz), *θ*_PV_ is exactly or almost equal to *θ*_1_ or *θ*_2_. As we shall see later, direction judgment in the identification task is also based on the population-vector analysis. Thus, the network uses the same readout scheme in both tasks.

The probability of a correct choice on any trial is determined by the percentage of trials on which the winning pool matches the one with a greater stimulus input. Figure 4B shows the psychometric function describing the probability of correct choices versus motion strength. The performance varies from chance to perfect discrimination when *c*′ is increased from 0% to 51.2%. The data are fitted by a Weibull function [47]:
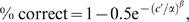
where *α* is the coherence level at which the performance is 82% correct and *β* describes the slope of the psychometric function. Our data are fitted by *α* = 6.85% and *β* = 1.45, consistent with the experimental values of 6.82% and 1.45 [13].

Figure 4C depicts the time course of population firing rates *r*_1_ and *r*_2_ averaged over correct and error trials, respectively. Given the coherent motion direction of *θ*_1_, the stimulus input to the pool selective for *θ*_1_ is larger than that to the other pool selective for *θ*_2_. On both correct and error trials, after the visual signal reaches the decision circuit, one pool ramps up it activity and thus ultimately wins the competition, whereas the other ramps down its activity. The population activity for the winner is lower on error trials than on correct trials, while that for the loser is less depressed on error trials. Furthermore, the ramping activity is more gradual on error trials. These differences become increasingly significant at higher coherence levels. This is because the winning neural pool receives less input on error trials than on correct trials, whereas the losing neural pool receives greater input on error trials than on correct trials. These trends have been observed experimentally in LIP activity (cf. Figure 11 in [13]).

**Figure 5:**
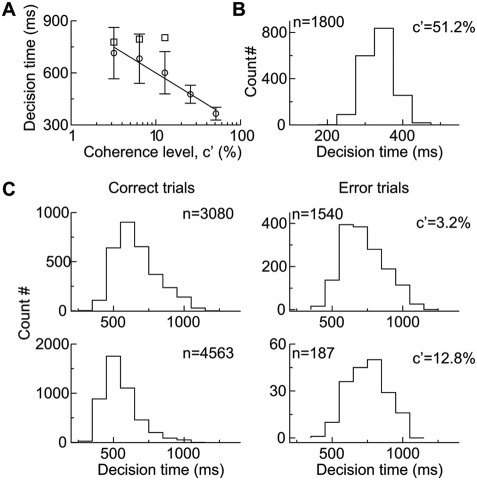
Decision time in the direction discrimination task. (A) Mean decision time as a function of motion strength. The mean decision time on error trials (square) is longer than that on correct trials (circle). The solid line is a linear fit to the data (circle). Error bars indicate SD. (B) The decision time histogram for *c*′ = 51.2% with the binwidth of 50 ms. (C) The histograms of decision time (with the binwidth of 100 ms) on correct (left) and error (right) trials for *c*′ = 3.2% (top) and 12.8% (bottom), respectively. Decision times are more variable at lower coherence levels. The number of trials used for plotting the histograms are indicated in the panels.

In the reaction-time version of the direction discrimination task, the decision time is measured as the time it takes for either of the two population firing rates to first reach a prescribed firing threshold (see Methods). This is in line with the observation that when a saccadic response is triggered, the up-ramping activity of LIP neurons reaches a stereotypical level that is independent of coherence level [13],[35]. The generation of saccadic motor responses is not explicitly modeled here. At each coherence level, the sum of the mean decision time and a fixed non-decision time (about 70 ms) is comparable with the experimentally measured reaction time (Figure 5A). In addition, the mean decision time decreases nearly linearly with *c*′ on a logarithmic scale, in agreement with the behavioral data [13]. Consistent with the population activity shown in Figure 4C, the mean decision time is longer on error trials than on correct trials. Note that the shape of the histogram for decision time depends remarkably on coherence level (Figure 5B and 5C). At high coherence levels, decision times are narrowly distributed around a short time (Figure 5B). At lower coherence levels, the up-ramping neural activity is slower (Figure 3C), resulting in longer decision times and broader distributions (Figure 5C). Decision times are more variable on error trials (right panels) than on correct trials (left panels). Thus, our model reproduces salient features of reaction times observed experimentally [30].

### Veridical Identification of Motion Direction

We have shown that a continuous recurrent network model reproduces salient experimental observations in the direction discrimination task [13],[35]. Now we turn to explore whether this circuit model also subserves analog computations underlying veridical judgments about motion direction. The simulations used the task protocol as in [4]. A random-dot motion stimulus was presented for a fixed duration of 1 s, followed by a saccadic eye movement indicating the monkey's judgment. On some trials, electrical stimulation was simultaneously applied to MT neurons for 1 s, and its impact on the monkey's direction estimates was measured. In this task, the monkey had the complete freedom to report veridically its perceived direction of motion in the visual stimulus. This judgment can be drastically different from the stimulus direction (*θ*_1_) since microstimulation may bias it toward the preferred direction (*θ*_2_) of MT cells at the microstimulation site. The generation of saccadic eye movements is not explicitly modeled.

### Neural Integration of the Visual Stimulus and the Electrically Elicited Directional Signal

**Figure 6:**
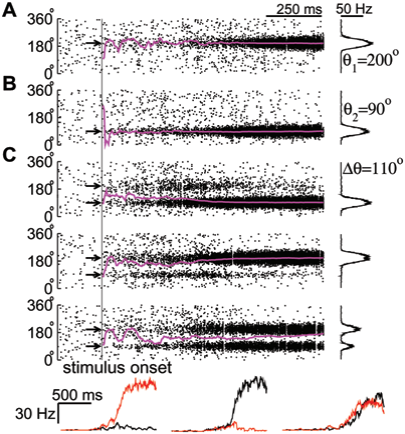
Neural activity related to direction identification in a veridical judgment task. (A) Neural response to the motion stimulus alone. (Left) Spatiotemporal firing pattern of pyramidal cells superimposed by the time course of the population vector (magenta). The arrow indicates the coherent motion direction (200°) of the stimulus. The motion stimulus is presented at 500 ms and lasts 1 s. (Right) Network activity profile at stimulus offset. The firing rate is calculated by counting the number of spikes fired by each neuron within 50 ms preceding the stimulus offset, divided by 50 ms. (B) Neural response to the microstimulation of MT neurons alone. The black arrow marks the microstimulated direction (90°). Same conventions as in (A). (C) Neural response to the simultaneous presentation of the motion stimulus and microstimulation. (Top three panels) Neural activity on three sample trials. (Bottom panels) Time course of population firing rates of two neural pools separately centered at 90° (red) and 200° (black), corresponding to the above three individual trials (from left to right).

Figure 6A depicts typical network activity in response to only a motion stimulus with *c*′ = 80% and *θ*_1_ = 200°. Before stimulus presentation, pyramidal cells exhibit low spontaneous activity, which is homogeneous across the population. After stimulus onset, a bell-shaped activity pattern develops around *θ*_1_ since the cells with preferred directions around *θ*_1_ are most activated. The network dynamics are reflected in the time course of the population vector *θ*_PV_, which converges to *θ*_1_ after initial transients (*magenta trace*). That is, the stimulus direction can be read out based on the population vector. If only microstimulation is applied to MT cells around *θ*_2_ (90°), a bump pattern develops and is centered at *θ*_2_ (Figure 6B). At the stimulus offset, active neurons show high firing rates comparable to those in Figure 6A, but the network activity profile is narrower. This results from the assumption that microstimulation activates a smaller number of MT neurons while MT neurons are widely tuned to visual stimuli. These results indicate that the network can represent directional signals by a bump state and that the population vector is a good measure for the network's direction judgments.

When both the visual stimulus and microstimulation are applied simultaneously, the input profile is bimodal with two peaks around *θ*_1_ (200°) and *θ*_2_ (90°) (cf. Figure 2C). Figure 6C displays the network activity on three trials. Owing to noisy input and stochastic neural dynamics, the network activity varies from trial to trial. On the first trial, one bump develops, and *θ*_E_, the value of *θ*_PV_ at stimulus offset, approximately equals *θ*_2_; that is, the direction estimate corresponds to the microstimulated direction. On the second trial, a single bump develops with *θ*_E_≃*θ*_1_, and hence the estimate corresponds to the stimulus direction. On the third trial, the network activity profile remains bimodal, and the value of *θ*_E_ is a weighted sum of two coexisting bumps. In this particular example, *θ*_E_ equals 174°, closer to the stimulus direction than to the microstimulated direction.

The model network integrates external inputs in the form of slow ramping activity, as if the motion stimulus and microstimulation provide conflicting evidence for direction judgments. This can be seen in the time course of population firing rates, *r*_1_ and *r*_2_, of the two neural pools separately centered at *θ*_1_ and *θ*_2_ (Figure 6C, bottom). On the first and second trials, *r*_1_ and *r*_2_ first ramp up together and then begin to diverge at a time that varies considerably from trial to trial. After the diverging point, one further ramps up, while the other ramps down. On the third trial, *r*_1_ and *r*_2_ remain comparable with *r*_1_ slightly larger than *r*_2_, consistent with the fact that the direction estimate is closer to the stimulus direction. Therefore, even when the motion strength is as high as 80%, the network behavior can be drastically distinct on different trials. This implies that the integration process is essentially stochastic. Moreover, here direction estimates are based on the profile of network activity, i.e., population averaging. If we instead used a scheme in which direction estimate is assigned by the preferred direction of the most active neuron, it would always be around either *θ*_1_ or *θ*_2_, inconsistent with the behavioral data [4].

### Effect of Microstimulation on Direction Judgments

**Figure 7:**
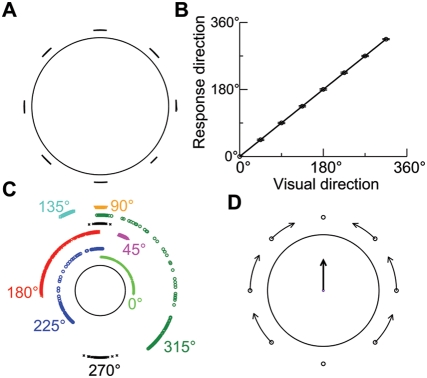
Effect of microstimulation on direction judgments. (A–B) Direction estimates (*θ*_E_) in the presence of motion stimulus alone. (A) The distribution of direction estimates on a ring for eight stimulus directions spanning 360° at 45° intervals. (B) The mean direction estimate versus the stimulus direction. The unity slope diagonal represents perfect identification performance on the task. Error bars indicate SD. (C,D) Direction estimates in the presence of both the motion stimulus and microstimulation. (C) The distribution of direction estimates on a ring for eight motion stimuli. Points are staggered radially for visualization purposes. (D) The shift of the mean direction estimate away from the stimulus direction (represented by open circle) due to the microstimulation of MT. The lines and arrows show the amplitude and direction of the shift in the mean direction estimate caused by microstimulation. The black arrow in the center denotes the overall effect of microstimulation on direction estimates, which is also the microstimulated direction.

As mentioned above, microstimulation can bias the direction identification. Here, we systematically change the stimulus direction (*θ*_1_) to explore the effect of microstimulation (with fixed *θ*_2_) on direction judgments. With the protocol as in [4], a motion stimulus is presented at 80% coherence with its coherent motion direction in one of eight directions spanning 360° in 45° increment. In the absence of microstimulation, the profile of network activity is peaked at *θ*_1_, and thus *θ*_E_ is around *θ*_1_. Figure 7A displays the distributions of *θ*_E_ values on a circle for eight different stimuli. In each case, the data points cluster densely with little variability. The mean value of *θ*_E_ accurately matches the stimulus direction, and the standard deviations are negligible (Figure 7B). Therefore, the network judges the stimulus direction very accurately.

When microstimulation is applied simultaneously with *θ*_2_ = 90°, the resulting distribution of *θ*_E_ values depends on the angular difference between the two stimuli, Δ*θ* = |*θ*_2_−*θ*_1_| (Figure 7C). Qualitatively, three types of effects can be distinguished. First, for a small Δ*θ* (e.g., 45° with *θ*_1_ = 45° or 135°), direction estimates from individual trials spread out between *θ*_1_ and *θ*_2_. Second, for an intermediate Δ*θ* (e.g., 135° with *θ*_1_ = 225° or *θ*_1_ = 315°), the distribution of *θ*_E_ values is discontinuous; most estimates cluster around either *θ*_1_ or *θ*_2_, but other estimates scatter between the two directions. Third, for a large Δ*θ* (e.g., 180° with *θ*_1_ = 270°), the distribution of *θ*_E_ values is bimodal, narrowly centered at *θ*_1_ and *θ*_2_.

Figure 7D depicts the shift of the mean value of *θ*_E_ away from the stimulus direction because of microstimulation, which can bias direction estimates toward the microstimulated direction. This effect occurs over nearly the whole range of stimulus directions (except for Δ*θ* = 0° or 180°). To show the overall effect of microstimulation, we calculated both the center-of-mass of all single-trial direction estimates in the absence of microstimulation and that in the presence of microstimulation. The black arrow in the center of Figure 7D denotes the direction of the vector from the nonstimulated to the stimulated center-of-mass, which is just the microstimulated direction.

### Mixed Strategy of Winner-Take-All and Vector Averaging

**Figure 8:**
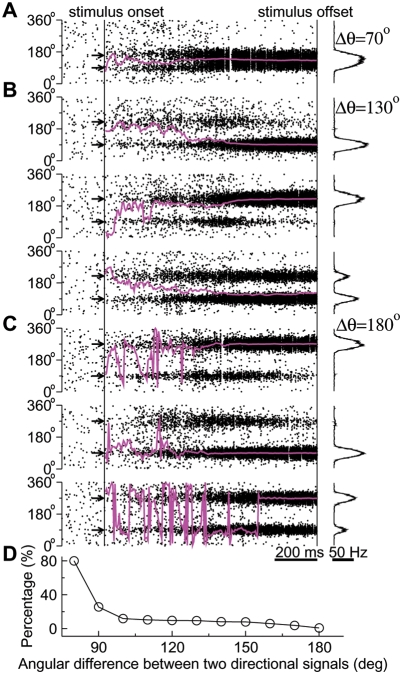
Distinct behavioral regimes during the probabilistic estimation of motion direction. Network activity can be distinguished based on the difference between the stimulus and microstimulated directions, Δ*θ*. Spatiotemporal firing pattern is superimposed by the time course of the population vector *θ*_PV_ (magenta). The network activity profile at the stimulus offset is shown on the right. The microstimulated direction is always 90°, while the stimulus direction *θ*_1_ varies with trials. (A) When Δ*θ* is relatively small (*θ*_1_ = 160°), direction estimates are based on vector averaging. (B) For an intermediate Δ*θ* (*θ*_1_ = 220°), the network exhibits winner-take-all on some trials (top and middle) and vector averaging on other trials (bottom). (C) For a large Δ*θ* (*θ*_1_ = 270°), network activity is predominated by the winner-take-all mechanism. (D) The percentage of trials on which the smaller of |*θ_E_*−*θ*_1_| and |*θ_E_*−*θ*_2_| (with *θ_E_* being the direction estimate) is larger than 10° as a function of Δ*θ*.

To understand the above three types of probabilistic direction identification, we investigated the network dynamics as Δ*θ* was systematically varied. When Δ*θ* is small, the input profile is unimodal, or there are two peaks but one is much shorter than the other (cf. Figure 2C, black trace with Δ*θ* = 45°). Consequently, the network response is relatively simple, as illustrated in Figure 8A for Δ*θ* = 70°. The stimuli activate large number of pyramidal cells with preferred directions between *θ*_1_ and *θ*_2_, resulting in a unimodal activity profile peaked at ∼125°, which is the average of *θ*_1_ = 160° and *θ*_2_ = 90°. Therefore, direction judgments are based on vector averaging.

On the other hand, for Δ*θ* = 180°, the input profile consists of two independent peaks, and two disjoint neural pools are activated. Thus, the network initially exhibits a bimodal activity profile, but the two bumps compete against each other over time (Figure 8C). At stimulus offset, one of the two bumps wins, and *θ*_E_ is close to either *θ*_1_ or *θ*_2_ (on the first and second trials). On very few trials (15 among 1800 trials), two bumps are visible (on the third trial); nevertheless, *θ*_E_ is still close to either *θ*_1_ or *θ*_2_. In this sense, direction judgment is determined by winner-take-all for a great Δ*θ*.

For a broad range of intermediate Δ*θ* between 70° and 170°, the input profile has two peaks at *θ*_1_ and *θ*_2_, but their width and height are not identical. The interaction of a visual stimulus and an artificially elicited directional signal is different from the visual-visual interactions [42]. Figure 8B shows the network activity for Δ*θ* = 130°, similar to the case with Δ*θ* = 110° (Figure 6C). The network behavior evolves based on the winner-take-all competition on some trials, where *θ*_E_ is close to either *θ*_2_ (on the first trial) or *θ*_1_ (on the second trial). On the other trials, however, two bumps develop initially and are sustained across the trial, in which cases the direction estimate is determined by vector averaging (*θ*_E_ = 120° on the third trial). In other words, direction estimates stochastically switch between the values determined separately by the winner-take-all and vector-averaging mechanisms across trials.

We found that the percentage *P* of trials on which the direction identification results from vector averaging decreases with increasing Δ*θ* (Figure 8D). *P* is larger than 80% for Δ*θ* = 80°; but it quickly becomes smaller than 10% for Δ*θ*>100° and smaller than 5% for Δ*θ*>150°. Therefore, for a sufficiently large distance between the two directional signals, the winner-take-all mechanism predominates. This can be explained as follows. The two neural subpopulations selectively responsive to the two input signals are sufficiently separated, so that they do not overlap nor excite each other significantly through localized lateral excitatory connections. Their interaction is mostly through shared feedback inhibition that underlies the winner-take-all competition. Owing to trial-to-trial neuronal fluctuations, however, the net inhibitory interactions may be insufficient to suppress the activity of either subpopulation on some trials, in which cases the direction estimation is determined by vector averaging.

**Figure 9:**
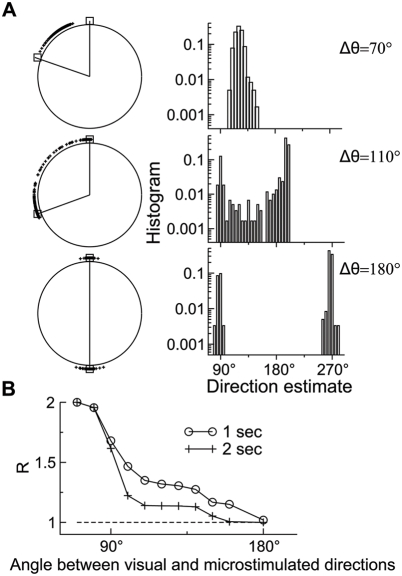
Winner-take-all versus vector averaging in direction identification. (A) The distribution of direction estimates on a circle (left) and the corresponding histogram with the binwidth of 5° (right). In each distribution, a wedge is defined by two directions (shown with open squares), separately denoting the median direction estimate for trials where the 80% coherence stimulus is applied alone and for trials where microstimulation is applied together with the 0% coherence stimulus. Three examples are displayed for Δ*θ* = 70°,110°, and 180°, respectively (from top to bottom). Six hundred simulations were performed for each case. (B) The index *R* as a function of the angular difference between the stimulus and microstimulated directions, Δ*θ*. Pure winner-take-all and vector averaging correspond to *R* = 1 and 2, respectively. The model displays a mixed strategy (with *R* between 1 and 2) for direction judgment over a wide range of Δ*θ* values. It also predicts that for a given intermediate Δ*θ*, a longer stimulus viewing time, for instance from 1 s (circle) to 2 s (cross), enhances the preponderance of the winner-take-all regime.

We further quantified the network's decision behavior by plotting the histograms of direction estimates (Figure 9A). For a small Δ*θ* such as 70°, all estimates lie between *θ*_1_ and *θ*_2_, and the histogram is approximately Gaussian-distributed. For an intermediate Δ*θ* such as 110°, most estimates are close to either *θ*_1_ or *θ*_2_, but there is also a substantial fraction of estimates in between. Accordingly, the histogram is bimodal. For a large Δ*θ* such as 180°, all estimates lie close to either *θ*_1_ or *θ*_2_, so that the histogram consists of two narrow and isolated peaks. These results confirm the above conclusion that the network's direction judgments are based on vector averaging when Δ*θ* is small, winner-take-all when Δ*θ* is large, and a mixture of both for intermediate Δ*θ* values.

*Nichols and Newsome* tested the winner-take-all versus vector-averaging coding schemes in the monkey experiment, using a measure called *R* that is defined as follows [4]. First, the median direction estimate is calculated separately for trials where the motion stimulus with *c*′ = 80% is presented alone (without microstimulation) and for trials where microstimulation is applied together with the 0% coherence stimulus. These two medians form a wedge (shown for our model in the left half of Figure 9A). *R* is then defined as the proportion of actual direction estimates (on the trials with both the 80% coherence stimulus and microstimulation) that lie within the wedge, divided by 0.5. As a result, *R* can be used to quantify the aforementioned three behavioral types. For instance, vector averaging implies that direction estimates lie completely within the wedge, so that *R*≃1/0.5 = 2. On the other hand, for pure winner-take-all, direction estimates are centered around the two medians, so that *R*≃0.5/0.5 = 1. *R* as a function of Δ*θ* is plotted in Figure 9B (open circle). *R* is close to 2 for small Δ*θ*, whereas it approaches unity when Δ*θ* is close to 180°, similar to the experimental observation (Figure 6 in [4]). Moreover, there is a plateau at *R*≃1.35 for a range of intermediate Δ*θ* values, a feature also present in the monkey data, which indicates a mixture of the winner-take-all and vector-averaging mechanisms. Note that the *R* curve is quite similar to the *P* curve shown in Figure 8D. Therefore, both the two entirely different measures confirm the mixed strategy for direction identification over a wide range of intermediate Δ*θ* values.

We reasoned that when the sensory stimulus and microstimulation provide conflicting signals, time integration may be important to resolve the ambiguity. In neuronal terms, a longer stimulus viewing time should allow one of two bumps in the network activity pattern to evolve to become dominant at the expense of the other. We tested this prediction by computing *R* under the condition where the motion stimulus lasted 2 s instead of 1 s. Indeed, with a longer stimulus viewing time, *R* generally becomes lower and is smaller than 1.15 when Δ*θ*≥110° (Figure 9B, cross). This model prediction is testable in future experiments.

## Discussion

Growing evidence indicates that in a random-dot motion discrimination task, while MT neurons encode motion directions, perceptual decisions are made downstream, perhaps in the parietal cortex [10],[12],[13],[35],[48],[49] or the prefrontal cortex [17]. Similarly, in a detection task (that requires a ‘yes or no’ binary response) using near-threshold somatosensory stimuli, neural activity in the prefrontal cortex, but not in the primary somatosensory cortex, was found to covary trial-by-trial with the subjective report [50]. What are the microcircuit properties that allow a ‘decision circuit’ to subserve perceptual judgments? We have previously proposed a cortical circuit model endowed with slow reverberatory excitation and feedback inhibition, which allows for the temporal integration of sensory stimuli and the formation of categorical choice [19],[21],[23]. This type of model framework has also been applied to somatosensory discrimination [18],[20],[24] and detection [25]. In the present study, we extended this approach to a continuous recurrent network. Our results suggest that a common cortical circuit can perform both the categorical discrimination and veridical judgment tasks.

### Temporal Integration and Categorical Choice in the Discrimination Task

In a two-alternative direction discrimination task, a subject must be instructed what are the discrete choice options by visual targets [13]. In the continuous recurrent network model, we implemented the two targets (at *θ*_1_ and *θ*_2_) and examined how the network integrates a motion stimulus biased by the targets and makes a categorical choice (*θ*_1_ or *θ*_2_). In consonance with the previous models with discrete neural pools [19],[21], our model reproduces salient observations of LIP activity in the monkey experiment [13]. First, the population firing rates of two competing neural pools first increase together and then diverge, with one continuing to build up while the other decaying down. Second, cells exhibit ramp-like activity, which is slower at lower motion strength. Third, the activity of the winning pool is higher on correct trials than on error trials, whereas the opposite is true for that of the losing pool. Furthermore, at the behavioral level, our model reproduces the psychometric and chronometric functions as well as the observation that the mean reaction time is longer on error trials than on correct trials [13].

In our model, slow temporal integration is instantiated by reverberatory excitation mediated by NMDARs [19],[21]. This is mainly related to its slow synaptic kinetics. We further tested this mechanism by partially replacing NMDARs with much faster AMPARs at recurrent excitatory synapses. As a result, the network's ability to integrate input signals is significantly reduced and the network's performance also deteriorates (data not shown). Experimentally, it would be interesting to measure whether direction discrimination becomes more impulsive and less accurate when NMDAR antagonists are applied to LIP in behaving monkeys. On the other hand, other slow positive feedback processes, such as short-term synaptic facilitation and those involving specific ion channels, could also contribute to time integration, which remains to be investigated experimentally and theoretically. In sum, we suggest that strong reverberation in a cortical microcircuit should be slow in order to subserve cognitive-type computations.

Recurrent excitation must be balanced by feedback inhibition [34],[51]. Lateral inhibition between neural pools involved in decision computation is consistent with the observation that the microstimulation of one neural pool in LIP not only speeds up the choices in its preferred direction but also slows down the choices in its null direction [49]. *Ditterich* found that an accumulator model produces reaction time distributions with long right tails, inconsistent with the behavioral data, and that the inclusion of lateral inhibition worsens the problem, resulting in even longer right tails especially at low coherence levels [30]. This is not the case in our model; the decision time distributions, although not Gaussian-distributed, do not show pronounced right tails, similar to those observed experimentally [30]. A distinguishing feature of our nonlinear network model is strong recurrent excitation, which is absent in linear accumulator models. The positive feedback mechanism ultimately leads to an acceleration of ramping neural activity toward a decision bound, preventing excessively long decision times. Indeed, *Ditterich* showed that the monkey's reaction time distributions can be well fitted by the accumulator model with an additional assumption that the decision bound decreases over time. This is functionally equivalent to a temporally increasing ramping slope, which naturally occurs in our recurrent circuit model.

### Mixed Strategy for Probabilistic Estimation of an Analog Stimulus Feature

We also applied the continuous recurrent network model to a direction identification task [4], assuming that the network represents a cortical area like LIP, downstream from MT. In the absence of physiological data, we assumed for the sake of simplicity that the inputs separately representing the motion stimulus and the electrically evoked directional signal sum linearly before being fed into the decision circuit. We also took into account lateral inhibition in MT [52],[53], assuming that the input profile for microstimulation has a Mexian-hat shape, which represents a nonlinear effect.

Since MT neurons are broadly tuned to visual motion signals, an important issue is how to link MT activity profile to subjects' percept. A number of studies have explored decoding strategies that the brain might use when there are two coexisting competing signals, each activating a different pool of MT neurons [42],[54],[55]. *Nichols and Newsome* inferred from the monkey's behavioral performance that different decoding schemes might be used when the angular distance between the direction signals is smaller or larger than 140° [4]. MT neurons with nearly opposite direction preferences appeared to compete to determine the monkey's percept, as predicted by winner-take-all; whereas MT neurons with preferred directions as different as 140° could cooperate to influence the monkey's percept, consistent with vector averaging or other distributed coding.

In our decision circuit, which is downstream from MT, direction judgments are based on the activity of all neurons. That is, we always use the population vector for direction estimation, and such estimates are in good agreement with the behavioral data. Nevertheless, when the stimulus and microstimulated directions are separated by a sufficiently large distance, direction judgments naturally instantiate winner-take-all, whereas when they are close to each other, direction judgments are consistent with vector averaging.

Interestingly, for the two directions with an intermediate angular distance, the network displays a “mixed strategy”, i.e., perceptual estimates are produced by winner-take-all on some trials and by vector averaging on the other trials. A prediction is that within this mixed-strategy regime, quick responses are based on vector averaging, whereas a longer integration of conflicting signals is more likely to yield a winner-take-all based categorical choice. Such temporal tradeoff should be observable at the level of neural activity. These specific model predictions can be tested in future experiments.

### Readout of Direction Judgments by Neurons Downstream from LIP

In the present work, we used a simple method (i.e., the population-vector analysis) to read out a direction estimate on each trial. In the future, it would be worthwhile to explicitly examine the neural circuit mechanism underlying the readout process. While cortical areas like LIP may be critically involved in accumulating information and making choices, the actual saccadic response that signals the monkey's decision is produced downstream. For instance, neurons in the superior colliculus, a command center for saccadic eye movements, respond to both the targets and the random-dot motion stimulus in the direction discrimination task [56]. It has been proposed that burst firing of movement neurons in the superior colliculus may be triggered when the synaptic excitation from ramping cortical neurons exceeds a threshold, thereby providing a cellular basis for a decision bound [22]. It will be worth exploring whether the superior colliculus circuit provides additional mechanisms that contribute to readout of perceptual decisions.

In fact, we have already developed an extended model in which a second circuit (that mimics the superior colliculus) receives synaptic input from the decision circuit and can generate a burst of activity signaling a saccade. This is essentially a continuous network version of the cortico-superior colliculus model (with four discrete neural pools) [22]. In this double-ring model, it is natural to read out direction estimates without assuming the threshold crossing of neural firing rates. Preliminary data (not shown) suggest that this extension does not significantly alter the conclusions drawn in this paper.

### Comparison with Other Models

It is worth noting that the continuous recurrent network model is adequate for the simulation of two perceptual decision tasks. In both tasks, the decision is about the coherent motion direction, which is a one-dimensional feature. In our network, each neuron has a preferred motion direction to which it is most sensitive. When the readout of direction judgments is based on population vector, downstream neurons will pool the activity of LIP neurons to produce a directional signal for saccadic eye response. Compared with the previous spiking network models on perceptual discrimination [19],[21], which have discrete (usually two) neural pools rather than a continuous network like ours, our work represents a distinct advance in the field. It would be rather straightforward to extend this one-dimensional model to a two-dimensional network model. For example, a two-dimensional firing-rate model for saccadic action selection (not perceptual decisions) has been proposed in [57]. However, computer simulations of such spiking neural circuits are computationally costly, especially for stochastic decision tasks where thousands of trials are required to gather necessary statistics under each condition (just as in the monkey experiments).

In this work, we have focused on the reaction-time version of the categorical discrimination task, in which a simulated trial is terminated when either of two population firing rates first reaches a threshold, and the corresponding choice and decision time are recorded. In the direction identification task, the response signaling a veridical judgment is produced at the offset of the visual stimulus presentation, as in the experiment of *Nichols and Newsome* [4]. Neither of the task paradigms involves working memory, and we did not specifically simulate the fixed-duration version of the discrimination task [12],[13].

While our model was based on that designed for spatial working memory [34], we changed some parameter values to reproduce comparable behavioral data from the monkey experiments (such as the psychometric and chronometric functions for the discrimination task and the *R* plot for the identification task). Interestingly, with this new set of parameter values, the network does not exhibit self-sustained persistent activity. This is at variance with our previous work using a model with discrete neural pools [19],[21]. In the future, it would be interesting to use the same model to simulate the fixed-duration version of the categorical discrimination task (where two targets exist throughout the trial) and analyze systematically to what extent the ability to carry out decision computation depends on the working memory capacity in the continuous network model (as we have previously done with the discrete model [21]).

### A Cognitive-Type Cortical Circuit Capable of Performing Multiple Functions

A continuous recurrent network model, which was originally developed for mnemonic delay-period activity in spatial working memory [34], has been elaborated in several ways [58]–[61]. Direction-selective persistent neural activity has been observed in both the prefrontal [62] and the posterior parietal cortex [63]. We argue that a cognitive-type cortical circuit like the parietal or prefrontal cortex is equipped with strongly recurrent connectivity to subserve both internal representation of information and dynamic decision computations. On the other hand, it is still unclear to what extent a network's capacity of decision computations and that of working memory necessarily depend on each other. Conceivably, top-down control signals could adaptively modulate a cortical circuit such as LIP, so that it can operate in different dynamical regimes to fulfill different computational demands. Regardless, the present work, by demonstrating that a single cortical circuit is able to perform the veridical judgment and categorical discrimination tasks, represents a significant step toward uncovering the circuit and neurodynamical underpinnings of cognition.
